# Alteration in the number, morphology, function, and metabolism of erythrocytes in high-altitude polycythemia

**DOI:** 10.3389/fphys.2024.1359357

**Published:** 2024-02-15

**Authors:** Song Yu, Yi Ye, Tana Wuren, Hai Yi

**Affiliations:** ^1^ College of Medicine, Southwest Jiaotong University, Chengdu, China; ^2^ Department of Hematology, Affiliated Hospital of Southwest Jiaotong University, The General Hospital of Western Theater Command, Chengdu, China; ^3^ Research Center for High Altitude Medicine, Qinghai University, Xining, China

**Keywords:** high-altitude polycythemia, erythrocyte, morphology, hematopoietic function, metabolism, 2,3-BPG

## Abstract

**Introduction:** High-altitude polycythemia (HAPC) is a common chronic high-altitude disease characterized by significantly increased erythrocyte, hemoglobin (Hb), and hematocrit values and decreased arterial oxygen saturation. The mechanisms underlying HAPC development are unclear; we aimed to investigate this in an HAPC rat model.

**Methods:** Twelve Sprague–Dawley rats were divided into control and HAPC groups. The HAPC group was exposed to hypobaric hypoxia. This HAPC model was assessed using routine blood tests and blood gas analyses. Bone marrow, peripheral blood reticulocytes (RETs), and peripheral blood erythrocyte apoptosis were measured using flow cytometry. Erythrocyte osmotic fragility (EOF) tests were conducted. Abnormal erythrocytes were counted using electron microscopy. Plasma-free hemoglobin, 5′-nucleotidase (CD73), adenosine, erythrocyte cytosolic adenosine, sphingosine-1-phosphate (S1P), and 2,3-bisphosphoglycerate (BPG) levels were measured using enzyme-linked immunosorbent assays. Erythrocyte metabolic pathway-related protein [adenosine A2B receptor (ADORA2B), erythrocyte equilibrative nucleoside transporter 1 (eENT1), sphingosine kinase 1 (SPHK1), phospho-SPHK1, bisphosphoglycerate mutase (BPGM), and glyceraldehyde 3-phosphate dehydrogenase (GAPDH)] levels were assessed by Western blotting.

**Results:** The HAPC rat model was successfully established (Hb > 210 g/L). Indices of bone marrow and peripheral blood RET proportions were significantly higher in the HAPC than the control group (*p* = 0.04 and *p* < 0.001, respectively). The proportion of peripheral blood erythrocytes in early apoptosis was significantly lower in the HAPC than the control group (*p* < 0.001). Vesicular erythrocyte and acanthocyte proportions were significantly higher in the HAPC than the control group (*p* < 0.001 and *p* = 0.019, respectively). The EOF tests revealed that 50% erythrocyte hemolysis occurred at 4.0–4.5 and 4.5–5.0 g/L NaCl in the control and HAPC groups, respectively. Plasma-free hemoglobin, CD73, adenosine, erythrocyte cytosolic adenosine, S1P, and 2,3-BPG levels and ADORA2B, eENT1, phospho-SPHK1, S1P, BPGM, and GAPDH erythrocyte expression levels (all *p* ≤ 0.02) were significantly higher in the HAPC than the control group.

**Conclusion:** In model rats, an HAPC-related erythrocyte increase was associated with enhanced bone marrow hematopoietic function and reduced erythrocyte apoptosis, whereas numerous abnormal erythrocytes, increased EOF, and reduced hemolysis resistance were associated with erythrocyte metabolism. CD73/adenosine/S1P/2,3-BPG and eENT1/adenosine/BPGM/2,3-BPG metabolic pathways in erythrocytes were activated in HAPC rats, facilitating oxygen release. These findings further reveal the intrinsic HAPC mechanism and forms a basis for future development of preventive and therapeutic strategies for HAPC.

## 1 Introduction

Erythrocytes are the most abundant and structurally simple nucleus-free cells in the blood. Despite their simplicity, they are vital for the survival of every cell in the body, as mature erythrocytes are responsible for oxygen transport. Erythrocytes are highly sensitive to hypoxia. Chronic hypoxia leads to a compensatory increase in erythrocytes as a response to such an environment, potentially leading to the development of high-altitude polycythemia (HAPC). Patients with HAPC exhibit significantly increased erythrocyte, hemoglobin, and hematocrit levels, along with reduced arterial oxygen saturation. Excessive erythrocyte proliferation can result in increased blood viscosity, microcirculatory disorders, and thrombosis and is also closely associated with pulmonary hypertension, myocardial infarction, and stroke (León-Velarde et al., 2005; Penaloza and Arias-Stella, 2007; Villafuerte and Corante, 2016). In 2004, the Sixth World Congress on Mountain Medicine and High Altitude Physiology in Qinghai established the international diagnostic criteria for HAPC (known as the Qinghai Criteria), i.e., hemoglobin concentration ≥210 g/L for males and ≥190 g/L for females (Jiang et al., 2014).

**FIGURE 1:**
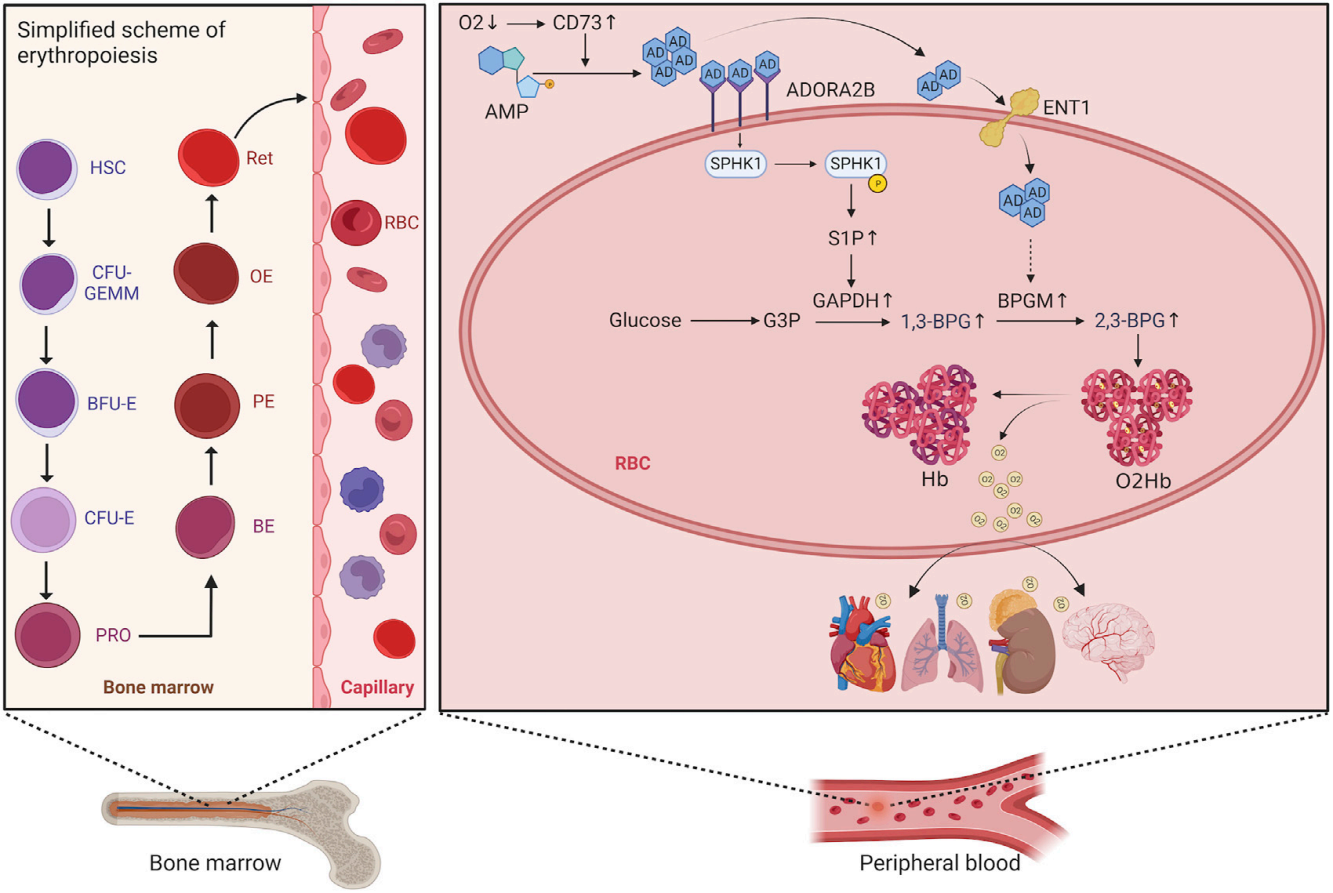
Schematic diagram of the erythropoietic process and red blood cell metabolism. HSC, hematopoietic stem cell; CFU-GEMM, colony-forming unit granulocyte, erythrocyte, monocyte, and megakaryocyte; BFU-E, burst-forming unit erythrocyte; CFU-E, colony-forming unit erythrocyte; PRO, proerythroblast; BE, basophilic erythroblast; PE, polychromatophilic erythroblast; OE, orthochromatophilic erythroblast; RET, reticulocyte.

Erythropoiesis consists of five stages, involving hematopoietic stem cells, progenitor cells, precursor cell proliferation and differentiation (from proerythroblasts to orthochromatic erythroblasts), reticulocyte (RET) proliferation and maturation, and release of mature erythrocytes from RETs into the peripheral blood. The process begins with hematopoietic stem cells differentiating into erythrocyte progenitor cells, further differentiation into burst-forming unit erythrocyte (BFU-E) cells, proliferation and maturation of these cells into colony-forming unit erythrocyte (CFU-E) cells, and then mitosis of these cells leading to differentiation into proerythroblasts, basophilic erythroblasts, polychromatic erythroblasts, orthochromatic erythroblasts, and finally into RETs with a nucleus. After further proliferation and maturation in the bone marrow, these RETs are transported to the peripheral blood, where they enucleate to mature into erythrocytes (Figure 1) (Bresnick et al., 2018). An increase in the proliferation and differentiation of erythroblasts and a decrease in apoptosis have been observed in the bone marrow of patients with HAPC (Wang et al., 2018; Ma et al., 2019). However, studies have reported increased apoptosis of erythroblasts in the bone marrow of mice under chronic hypoxic conditions (Yu et al., 2018). Generally, most studies suggest that bone marrow erythropoiesis is activated in HAPC.

The deformability of erythrocytes is primarily related to membrane properties and morphology (Brun et al., 2021), and reduced deformability of erythrocytes can easily result in hemolysis. In individuals who rapidly ascend to high altitudes, a significant number of abnormal erythrocytes are present. Moreover, hypoxia can affect erythrocyte deformability. However, whether the morphology and function of erythrocytes in HAPC are normal remains unclear. The morphological changes in these erythrocytes and their adaptation to hypoxia may be interconnected. Nonetheless, these changes could also adversely impact erythrocyte function.

Erythrocytes are both carriers and sensors of oxygen, and hypoxia inevitably affects erythrocyte metabolism. Recent evidence indicates that the molecular mechanisms underlying the regulation of erythrocyte metabolism under hypoxic conditions involve signaling in adenosine, sphingosine 1-phosphate (D’Alessandro et al., 2023), and glutamyltransferase-dependent pathways, and that hypoxia also promotes erythrocyte glycolysis, deregulation of the pentose phosphate pathway, as well as purine catabolism, glutathione homeostasis, arginine/nitric oxide, and sulfur/H2S metabolism (D’Alessandro et al., 2016). Further, expression of human Rh blood group components may contribute to ammonium homeostasis by affecting intracellular pH, which in turn promotes BPGM activity and facilitates 2,3-BPG production (D’Alessandro et al., 2024).

Previously, our group identified metabolic abnormalities in erythrocytes of patients with HAPC and HAPC model mice. We observed a significant increase in intracellular sphingosine-1-phosphate (S1P, a biologically active signaling lipid) and 2,3-bisphosphoglycerate (2,3-BPG, a negative allosteric modulator of oxygenated hemoglobin [Hb-O_2_]) (Qile et al., 2023). Our goal was to enhance the understanding of metabolic changes in erythrocytes in HAPC and provide insights for future prevention and treatment strategies for the condition. Thus, in the present study, we aimed to further investigate the levels of RETs in the peripheral blood and bone marrow of HAPC model rats, examine the apoptosis and morphological changes of their peripheral blood erythrocytes, measure the osmotic fragility and hemolytic function of these cells, and further investigate the metabolic changes in erythrocytes.

## 2 Materials and methods

### 2.1 Animal grouping

Twelve specific pathogen-free male Sprague–Dawley (SD) rats were randomly divided into a control group and an HAPC group. For the HAPC rat model, HAPC rats were placed in a hypobaric hypoxic chamber (Guizhou Fenglei Oxygen Chamber, dyc-300; Guizhou Fenglei Oxygen Chamber Co., Ltd., Quizhou, China), simulating an altitude of 5,000 m, with the following conditions: atmospheric pressure, ∼50 kPa; oxygen concentration maintained at 10%; environmental temperature, 20°C–24°C; relative humidity, 40%–70%; 12-h/12-h light/dark cycle. The rats were kept under these conditions for 28 days. The control group rats were raised in specific pathogen–free-grade animal rooms, with the same temperature, humidity, lighting time, and feeding conditions as the HAPC group.

### 2.2 Collection of rat plasma and erythrocytes

After anesthetizing all SD rats with an intraperitoneal injection of pentobarbital sodium, blood from the abdominal aorta was collected into vacuum heparin tubes. The whole blood in the anticoagulant tubes was used for routine blood tests, blood gas analysis, erythrocyte osmotic fragility (EOF) experiments, and peripheral blood RET detection. For plasma collection, the remaining anticoagulated whole blood was centrifuged at 4°C and 2,500 rpm for 10 min, and the supernatant plasma was aliquoted into 200-μL samples for subsequent enzyme-linked immunosorbent assay (ELISA) detections. For erythrocyte collection, after collecting the plasma, the layer with white blood cells was discarded from the anticoagulant tubes, and erythrocytes in the bottom layer were resuspended in 2 mL of 1× Tris-buffered saline (TBS), centrifuged at 4°C and 2,500 rpm/min for 5 min to remove the supernatant, washed twice, then the washed erythrocytes were kept for later use at 4°C.

### 2.3 Collection of bone marrow cells of rats

After blood collection, the rats were euthanized by decapitation, and bilateral femurs were removed. Bone marrow was harvested by repeatedly flushing the marrow cavity with heparin/phosphate-buffered saline (PBS) using a 20-mL syringe and stored at 4°C in the dark for later examination.

### 2.4 Preparation and scanning of erythrocytes and peripheral blood erythrocyte morphology analysis with electron microscopy

TBS-washed erythrocytes (150 μL) were added to a 1.5-mL Eppendorf tube, and 1.5 mL of 1:5 diluted fixative (2.5% glutaraldehyde in 1 × PBS buffer) was slowly added along the tube wall to resuspend the cells. The cells were left to stand at 4°C for 5 min, then centrifuged at high speed (4°C, 12,000 rpm for 10 min). The supernatant was removed, and the sediment was retained. Then, 2.5% glutaraldehyde fixative was slowly added along the tube wall, and the sample was stored at 4°C.

Five hundred erythrocytes were counted and analyzed per sample. They were classified into two types based on morphology: normal or abnormal erythrocytes. The abnormal erythrocytes were further subdivided into acanthocytes (irregular serrated protrusions on the cell periphery), vesicular erythrocytes (irregular vesicular protrusions on the cell membrane), or severe abnormalities (both acanthocytic and vesicular erythrocyte changes in the same cell).

### 2.5 Protein extraction and cytoplasmic preparation of erythrocytes

The washed erythrocytes were mixed with a strong radioimmunoprecipitation assay (RIPA) buffer (50 μL RIPA + 1 μL phenylmethylsulfonyl fluoride + 1 μL phosphatase inhibitor) at a 1:1 ratio for lysis. The mixture was lysed on ice for 30 min, vortexed for 5 s every 10 min, and then centrifuged at 4°C at 12,000 rpm for 10 min. The supernatant was collected, boiled with 2× loading buffer for 10 min, aliquoted, and stored at −80°C as samples for Western blotting.

Additionally, 2 mL of washed erythrocytes were treated with four freeze–thaw cycles with liquid nitrogen, followed by centrifugation at 4°C at 12,000 rpm for 10 min, and the supernatant was collected as the cytoplasmic extract of erythrocytes.

### 2.6 Staining and analysis procedures for the detection of erythrocyte apoptosis in rat peripheral blood based on flow cytometry

A total of 10 μL of TBS-washed erythrocytes were added to 990 μL of 1 × PBS. Subsequently, 10 μL of this diluted erythrocyte solution was transferred to a fresh tube containing 990 μL of 1 × PBS, gently mixed, and centrifuged at 300 × g for 5 min. The supernatant was discarded, and the cells were resuspended in 100 μL of annexin V binding buffer (1×). To this suspension, 2.5 μL of annexin V–fluorescein isothiocyanate (FITC) reagent and 2.5 μL of propidium iodine reagent (50 μg/mL) were added. The mixture was gently vortexed and incubated at room temperature in the dark for 20 min. After incubation, 900 μL of annexin V binding buffer (1×) was added, and the sample was mixed and filtered for analysis.

For the analysis of the proportion of erythrocytes in early apoptosis, the specific gating strategy is shown in Supplementary Figures S1H–J. FITC+/PerCP/Cy5.5− represents erythrocytes in early apoptosis, and FITC+/PerCP/Cy5.5+ represents erythrocytes in late apoptosis or necrosis.

### 2.7 Staining and analysis procedures for the detection of RETs in rat peripheral blood based on flow cytometry

One milliliter of BD Retic-Count™ reagent and 1 mL of PBS were added to 5 μL of whole blood and incubated at room temperature in the dark for 30 min. Then, 100 μL of the stained solution was added to 1 mL of PBS, and the sample was filtered to be ready for analysis. The specific gating strategy is shown in Supplementary Figures 1F–G, with Flow Jo v10.8.1 (https://www.flowjo.com/solutions/flowjo/downloads/previous-versions) used for analysis and the fluorescence intensity of RETs used as the indicator.

### 2.8 Staining and analysis procedures for the detection of RETs in rat bone marrow based on flow cytometry

Bone marrow RETs were labeled with CD71-PE, leukocytes with CD45-FITC, and DNA with the nucleic acid dye DRAQ5 to establish a flow cytometry–based detection scheme for marrow micronuclei. Bone marrow cells were centrifuged at 300 × *g* for 5 min, and the supernatant was discarded. The cells were resuspended in 50 μL PBS, and 20 μL of the resuspension was added to 100 μL of the staining system (containing 2 μg CD71-PE, 2 μg CD45-FITC, and 2 μL FBS). After mixing well, the sample was left to stain in the dark at 4°C for 30 min. Following surface antigen staining, the bone marrow cells were transferred to a centrifuge tube containing 5 mL PBS for washing, and the tube was centrifuged at 300 × *g* for 5 min before the supernatant was discarded to remove free antibodies. The cell pellet was then resuspended in 1 mL of DRAQ5 staining solution (20 μmol/L), mixed well, and stained in the dark at 37°C for 30 min. After a further wash with 5 mL PBS and centrifugation (300 ×*g*, 5 min), the supernatant was discarded, and the cells were resuspended in 1,250 μL PBS, filtered, and transferred to a flow cytometry tube. Samples were stored in the dark at 4°C and analyzed by flow cytometry within 4 h (Zeng et al., 2020).

The specific gating strategy is shown in Supplementary Figures 1A–E, with Flow Jo v10.8.1 software used for analysis and the proportion of RETs used as the indicator. DRAQ5+/FITC + indicated RETs with micronuclei, DRAQ5−/FITC− indicated mature erythrocytes, and DRAQ5−/FITC + indicated RETs. The formula for calculating the RETs proportion (%) is (Q2 + Q3)/(Q1 + Q2 + Q3 + Q4) × 100, where Q1 represents red blood cells (RBCs) with micronuclei, Q2 represents RETs with micronuclei, Q3 represents RETs, and Q4 represents RBCs. The product numbers of all flow cytometry antibodies are presented in Supplementary Table S1.

### 2.9 ELISA detection of plasma free hemoglobin, CD73, S1P, adenosine (intracellular and extracellular), and 2,3-BPG

Plasma-free hemoglobin, plasma 5′-nucleotidase (CD73), and extracellular adenosine were detected using ELISA with plasma samples, whereas S1P, intracellular adenosine, and 2,3-BPG were detected using erythrocyte cytoplasmic extraction. All ELISA kits were provided by Nanjing Camilo Biological Co., Ltd. (Nanjing, China), and the procedures were performed according to the manufacturer’s instructions. The product numbers of all ELISA kits are presented in Supplementary Table S1.

### 2.10 EOF test

Adequate amounts of Parpart NaCl working solution were prepared at a Parpart NaCl solution/ddH_2_O ratio of 1:9. Heparin-anticoagulated blood was prepared for later use: 5-mL Eppendorf tubes were obtained and numbered sequentially (1–17), and Parpart NaCl working solution and ddH_2_O were added according to the Parpart NaCl dilution table, with a total volume of 4 mL (see Supplementary Table 2 for the Parpart NaCl dilution table). Next, 40 μL of heparin-anticoagulated blood was added to the above-described diluted Parpart NaCl solution, mixed immediately, and left at room temperature for 30 min. The tubes were then centrifuged at 300 × *g* for 5 min, and the supernatant was collected. The optical density at 540 nm was measured using a microplate reader, with the supernatant from the tube containing 8.5 g/L NaCl (tube 3) used as a blank and the tube containing 1 g/L NaCl (tube 17) used as a reference for 100% hemolysis, to calculate the percentage of hemolysis.

### 2.11 Western blot

Protein extracts were separated using 10% sodium dodecyl sulfate–polyacrylamide gel electrophoresis and then transferred to a transfer membrane (Millipore, Burlington, MA, United States). After blocking with 5% bovine serum albumin for 1 h, the membrane was incubated with primary antibodies overnight at 4°C. The antibodies used were as follows: anti-adenosine A2b receptor [ADORA2B]/ADORA2B antibody (ab229671, 1:500; Abcam, Cambridge, United States), sphingosine kinase 1 (SPHK1) polyclonal antibody (10670-1-AP, 1:500; Proteintech, Wuhan, China), phospho-SPHK1 (Ser225) rabbit polyclonal antibody (19561-1-AP, 1:500; Proteintech), ENT1 polyclonal antibody (PA5-75507, 1:500; Invitrogen, Waltham, MA, United States), bisphosphoglycerate mutase (BPGM) polyclonal antibody (17173-1-AP, 1:500; Proteintech), anti-glyceraldehyde 3-phosphate dehydrogenase (GAPDH) antibody (ab181602, 1:10,000; Abcam), and beta-actin rabbit polyclonal antibody (20536-1-AP, 1:1,000; Proteintech). The membrane was then incubated with the secondary antibody, i.e., horseradish peroxidase–conjugated affinity-purified goat anti-rabbit IgG (H + L) (SA00001-2, 1:5,000; Proteintech) at room temperature for 1 h. Finally, protein bands were visualized using SuperKine ultra-sensitive ECL substrate (BMU102-CN/BMU103-CN; Abbkine, Wuhan, China), and quantified using ImageJ software (NIH, Bethesda, MD, United States).

### 2.12 Statistical analyses

Statistical analyses were performed using SPSS v25.0 (IBM Corp., Armonk, NY, United States). The normality of data distribution was assessed using the Kolmogorov–Smirnov and Shapiro–Wilk tests, and the homogeneity of variances was evaluated using the F test. Data that were normally distributed and had homogeneity of variance were analyzed using two-tailed independent-samples *t*-tests; data that were normally distributed but had heterogeneity of variance were analyzed using a *t*ʹ test (data for the *t* and *t*ʹ tests are presented as mean ± standard deviation). Data that were not normally distributed were analyzed using the Mann–Whitney U test (data are presented as P50 [P25, P75]). GraphPad Prism v9.5.0 (GraphPad Inc., La Jolla, CA, United States) was used for plotting graphs. *p* < 0.05 was considered statistically significant.

## 3 Results

### 3.1 Successful establishment of an HAPC rat model

After placing the SD rats in a hypobaric hypoxic chamber for 28 days, blood was drawn from the abdominal aorta for routine blood tests and blood gas analyses. Rats in the HAPC group, which were exposed to chronic hypoxia, had significantly higher hemoglobin content, erythrocyte counts, and hematocrit values than rats in the control group (Table 1). The average hemoglobin content in the control group was 140.5 g/L, whereas the lowest in the HAPC group was 211 g/L, with an average of 237 g/L. The median erythrocyte count in the control group was 5.83 × 10^12^/L, compared with 9.41 × 10^12^/L in the HAPC group. The average hematocrit in the control group was 36.45% compared to 59.78% in the HAPC group. Additionally, arterial oxygen saturation and partial pressure of oxygen were significantly lower in the HAPC group than in the control group (*p* < 0.001). According to the Qinghai Criteria for chronic high-altitude disease, the HAPC model was thus successfully established in all rats.

**TABLE 1:** Results of routine blood and blood gas analysis in the control and HAPC groups.

		Control (*n* = 6)	HAPC (*n* = 6)	t	Z	*p*
Routine blood examination	RBC Count (× 10^12/L)	5.83 (5.67, 6.14)	9.27 (8.35, 9.70)		−2.882	0.004
	HB (g/L)	140.5 ± 9,87	239.67 ± 19.63	−11.05		<0.001
	HCT (%)	36.45 ± 2.47	60.10 ± 3.53	−13.43		<0.001
	PLT (×10^9/L)	755.50 (636.00,778.50)	368.00 (199.75,526.25)		−2.326	0.02
	MCV (fL)	61.28 ± 0.79	66.00 ± 3.07	−3.647		0.012
	MCH (pg)	23.60 ± 0.47	26.25 ± 0.86	−6.587		<0.001
	MCHC (g/L)	385.33 ± 3.67	398.50 ± 12.37	−2.499		0.032
Arterial blood gas analysis	PO_2_ (mmHg)	67.50 ± 5.68	44.50 ± 3.02	8.756		<0.001
	SO_2_ (%)	93.33 ± 2.34	76.33 ± 4.03	8.932		<0.001

HAPC, high-altitude polycythemia; RBC, red blood cells; HB, hemoglobin; HCT, hematocrit; PLT, platelet; MCV, mean corpusular volume; MCH, mean corpusular hemoglobin; MCHC, mean corpusular hemoglobin concerntration.

### 3.2 Changes in erythrocyte count in HAPC rats

A significant increase in peripheral blood erythrocytes was observed in the HAPC group, consistent with the mechanism behind HAPC, which is believed to involve an imbalance in erythrocyte apoptosis/proliferation (Wu and Du, 2013). Therefore, we further investigated the content of RETs in bone marrow and peripheral blood as well as the apoptosis of peripheral blood erythrocytes.

#### 3.2.1 Increase in RETs in the bone marrow and peripheral blood of HAPC rats

**FIGURE 2:**
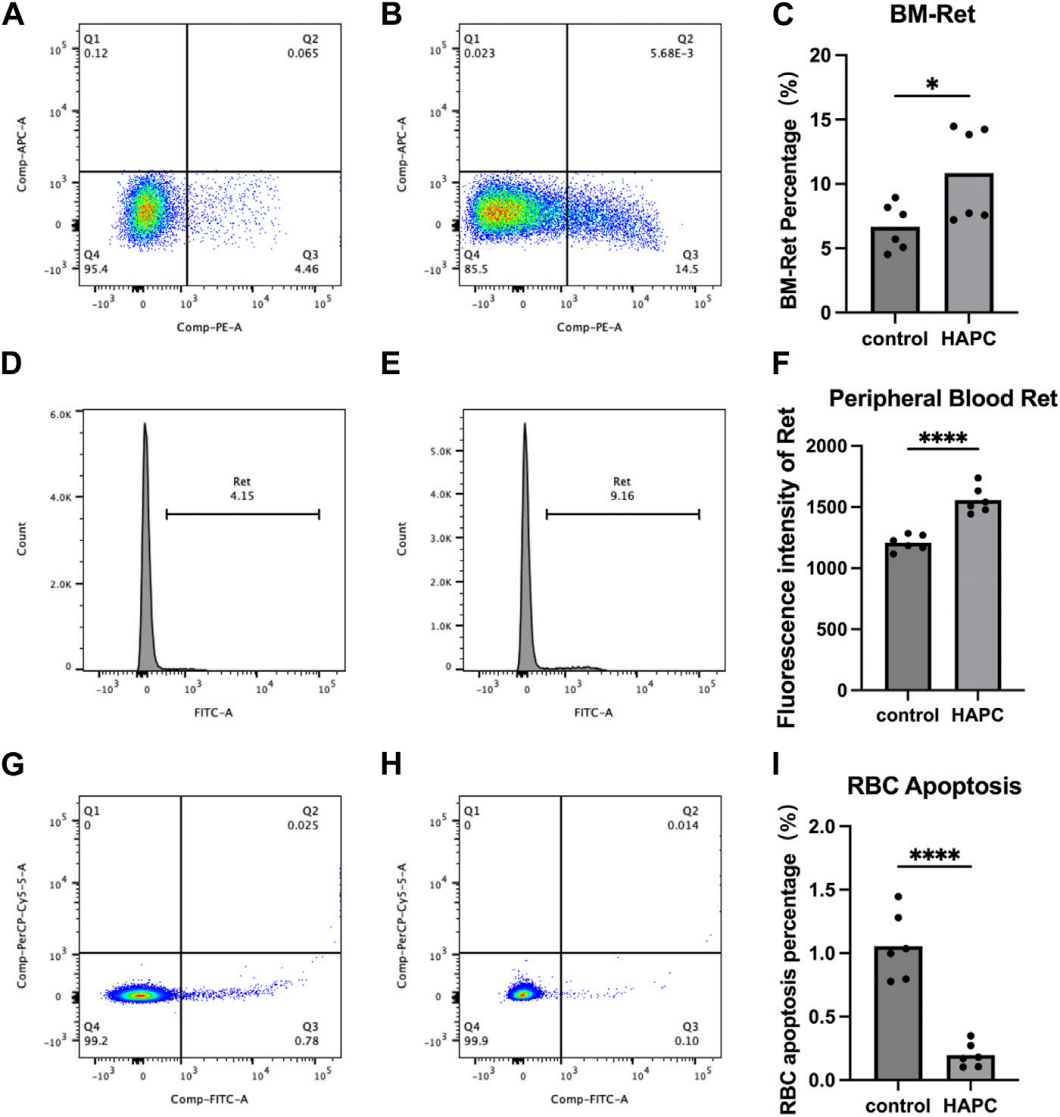
Flow cytometry analysis results. **(A–C)** Bone marrow reticulocytes: **(A)** control group, **(B)** HAPC group, and **(C)** statistical analysis of bone marrow reticulocytes by flow cytometry results. **(D–F)** Peripheral blood reticulocytes: **(D)** control group, **(E)** HAPC group, and **(F)** statistical analysis of peripheral blood reticulocytes by flow cytometry results. **(G–I)** Peripheral blood erythrocyte apoptosis: **(G)** control group, **(H)** HAPC group, and **(I)** statistical analysis of peripheral blood erythrocyte apoptosis by flow cytometry results. HAPC, high-altitude polycythemia.

In a rat model of chronic intermittent hypoxia, an increase in the number of pluripotent hematopoietic progenitor cells, which was suggested to promote erythropoiesis, was observed in the bone marrow (Alvarez-Martins et al., 2016). An increase in the number of megakaryocyte–erythrocyte progenitors in the bone marrow was also seen in hypoxic mice (Li et al., 2011), supporting the notion that the bone marrow hematopoietic system is activated under hypoxic conditions. The final maturation of erythrocytes requires the transfer of RETs from the bone marrow to the peripheral blood for enucleation and maturation into anucleate erythrocytes. Therefore, we used flow cytometry to detect the proportion of RETs in the bone marrow and fluorescence intensity to assess RETs in the peripheral blood. We found that the proportion of bone marrow RETs in the control group (6.68% ± 1.81%) was significantly lower than that in the HAPC group (10.84% ± 3.67%; *p* = 0.04) (Figures 2A–C). The fluorescence intensity of peripheral blood RETs in the HAPC group (1556.17 ± 109.87) was significantly higher than in the control group (1207.50 ± 64.38; *p* < 0.001) (Figures 2D–F). Under chronic hypoxia, the number of RETs (as precursors of mature erythrocytes) increased significantly, leading to an increase in erythrocytes. However, under normal conditions, proliferation/apoptosis of erythrocytes reaches homeostasis, and theoretically, apoptosis would increase as proliferation increases.

#### 3.2.2 Decrease in the apoptosis of peripheral blood erythrocytes of HAPC rats

A stable hematopoietic environment requires a balance between cell proliferation and apoptosis. Under hypoxic conditions, erythrocyte apoptosis is reduced during erythropoiesis, which leads to erythrocytosis in mice under long-term hypoxia (Harada et al., 2015). Therefore, we explored the apoptosis of peripheral blood erythrocytes using flow cytometry. The early apoptosis rate of peripheral blood erythrocytes in the control group was 1.06% ± 0.27%, whereas it was significantly lower in the HAPC group at 0.20% ± 0.10% (Figures 2G–I; *p* < 0.001). Our results also confirmed that, although the number of RETs indeed increased, the apoptosis rate of RETs in the peripheral blood decreased in HAPC rats, which jointly led to an increase in erythrocytes.

### 3.3 Increase in erythrocytes with morphological abnormalities in HAPC rats

**FIGURE 3:**
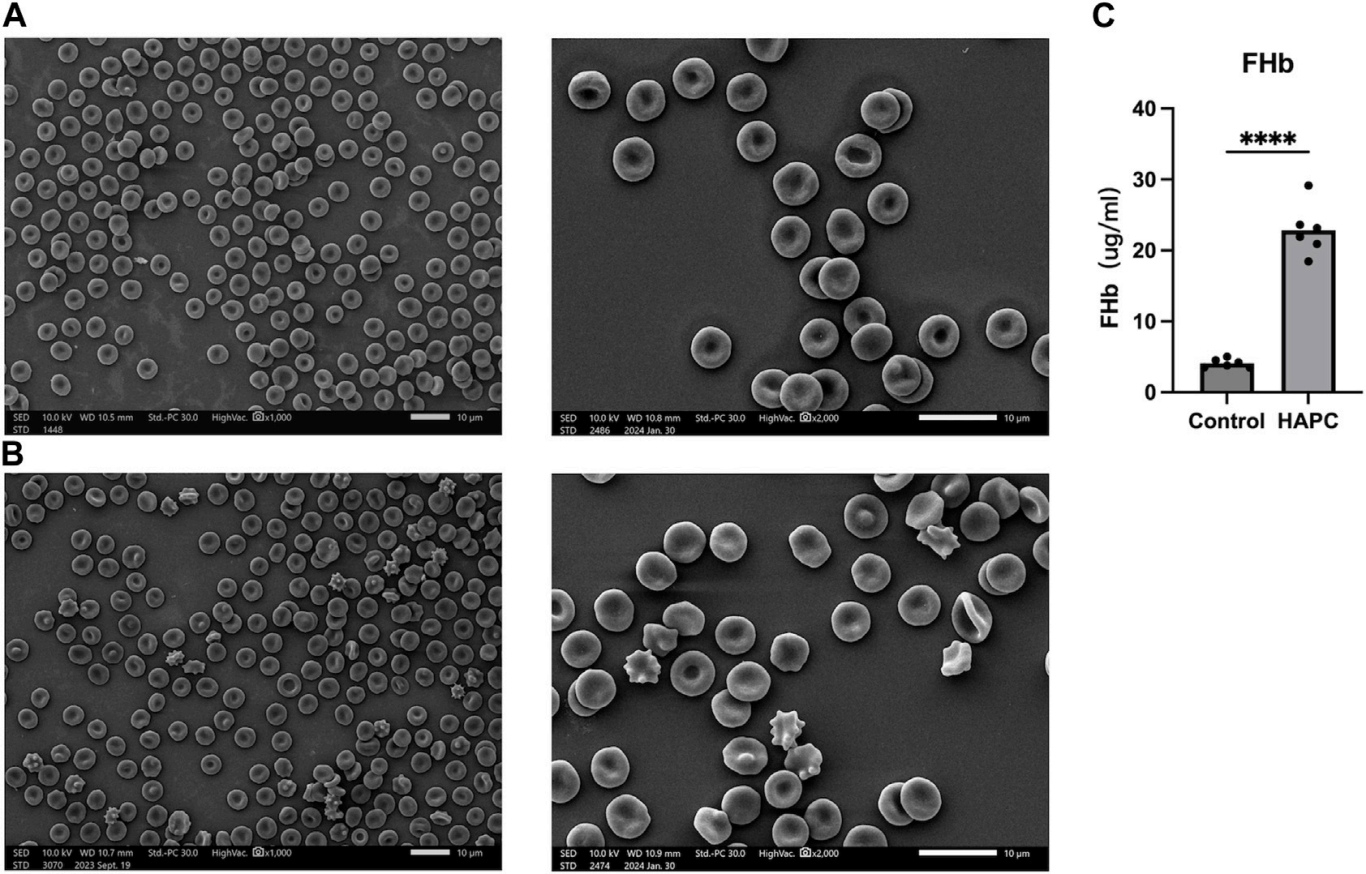
**(A)** SEM of peripheral blood erythrocytes in the control group, ×1000 (left), ×2000 (right). **(B)** SEM of peripheral blood erythrocytes in the HAPC group, ×1000 (left), ×2000 (right), and **(C)** statistical result of plasma FHb content. SEM, scanning electron microscopy; HAPC, high-altitude polycythemia; FHb, free hemoglobin.

Short-term hypoxia caused by a rapid ascent into high altitudes can increase the incidence of erythrocyte morphological abnormalities (Gu et al., 2017). Therefore, we used electron microscopy to investigate changes in morphology and the number of abnormal-shaped erythrocytes in chronic HAPC rats (Figures 3A, B). The number of normal erythrocytes was significantly higher in the control than the HAPC group (*p* < 0.001), with abnormal-shaped erythrocytes primarily being echinocytes and acanthocytes. The number of abnormal-shaped erythrocytes was significantly higher in the HAPC than the control group (vesicular, *p* < 0.001; acanthocytes, *p* = 0.019; severe abnormalities, *p* = 0.003) (Table 2).

**TABLE 2:** Comparison of the morphology and quantity of peripheral blood erythrocytes between control and HAPC rats (piece/portion).

	Control (*n* = 5)	HAPC (*n* = 6)	t	*p*
Normal form	461.20 ± 15.27	396.83 ± 16.46	6.667	< 0.001
Vesicular	18.80 ± 7.29	56.83 ± 11.20	−6.503	< 0.001
Acanthocytes	12.80 ± 6.38	25.33 ± 7.94	−2.84	0.019
Severe deformity	7.20 ± 2.78	21.00 ± 7.04	−4.095	0.003

### 3.4 Increase in EOF and hemolysis in HAPC rats

In rats with HAPC, both the number and morphology of erythrocytes underwent significant changes. Therefore, we further explored whether erythrocyte function was changed, with EOF and hemolysis being the main focus of our investigation. EOF is a measurement of the resistance of erythrocytes to hypotonic solutions and hemolysis, which primarily depends on the surface area/volume ratio in erythrocytes. Clinically, EOF can be applied to screening and evaluating certain hemolytic diseases, such as thalassemia, hereditary spherocytosis, and glucose-6-phosphate dehydrogenase deficiency (Emilse et al., 2018; Tatu and Sweatman, 2018). Routine blood tests showed that the average erythrocyte volume in HAPC rats had increased, leading to a relative decrease in the surface area/volume ratio of erythrocytes, which may increase osmotic fragility. Our subsequent EOF experiments further corroborated this theory. The 50% hemolysis rate for erythrocytes was 4.0–4.5 g/L NaCl in the control group and 4.5–5.0 g/L NaCl in the hypoxia group (Table 3). Long-term hypoxia led to a decrease in the resistance of erythrocytes to hypotonic solutions, increased osmotic fragility, reduced deformability, and decreased oxygen transport capacity in the HAPC group.

**TABLE 3:** Summary of hemolysis rates in the erythrocyte osmotic fragility test between the control and the HAPC groups.

NaCl (g/L)	Hemolysis ratio	sample	C1 (%)	C4 (%)	C5 (%)	C6 (%)	H1 (%)	H3 (%)	H5 (%)	H6 (%)
10			−0.1	−0.2	−0.2	0.2	0.1	0.2	−0.1	−0.2
9			0.0	−0.4	−0.8	−0.2	0.1	0.0	0.0	−0.6
8.5			0.0	0.0	0.0	0.0	0.0	0.0	0.0	0.0
8			0.2	0.0	−0.7	0.1	0.4	0.0	0.0	−0.5
7.5			0.0	−0.1	−0.2	−0.4	0.1	0.2	0.1	−0.4
7			−0.2	−0.4	−0.7	0.1	0.0	−0.1	0.0	−0.5
6.5			−0.1	−0.1	−0.2	0.4	0.1	0.0	0.3	−0.4
6			−0.2	−0.2	−0.7	−0.3	0.3	0.2	0.7	−0.5
5.5			0.0	−0.4	−0.7	−0.3	1.2	1.0	2.2	1.3
5			0.1	0.1	−0.1	0.5	10.3	10.6	25.1	22.2
4.5			17.0	18.1	21.4	28.9	62.0	59.0	61.8	60.5
4			71.2	74.3	80.5	83.3	92.0	86.6	86.2	73.8
3.5			86.4	94.4	95.6	98.4	99.1	93.1	92.2	101.5
3			87.7	95.6	97.7	98.6	93.8	96.5	86.9	95.0
2.5			89.5	97.6	99.8	98.9	97.5	100.0	87.5	99.0
2			90.2	97.0	95.6	97.2	92.3	95.4	95.9	94.2
1			100.0	100.0	100.0	100.0	100.0	100.0	100.0	100.0

The increased EOF and reduced resistance to hemolysis in HAPC rats led us to investigate hemolysis further. Previous evidence indicates that, under hypoxic stimulation, most extracellular ATPs are derived from the lysis of erythrocytes, and increased ATP levels are closely related to plasma-free hemoglobin, which primarily reflects intravascular hemolysis (Ellsworth et al., 1995; Sikora et al., 2014; Grygorczyk and Orlov, 2017). ELISA showed that the plasma free hemoglobin concentration was significantly higher in the HAPC group (22.87 ± 3.59 μg/mL) than the control group (4.09 ± 0.61 μg/mL, *p* < 0.001) (Figure 3C).

### 3.5 RBC metabolic pathway changes manifest RBC oxygen release in HAPC rats

Hypoxia can affect erythrocyte metabolism, such as adapting erythrocyte-mediated purinergic signaling to hypoxia (Sun et al., 2017), changing erythrocyte glycolysis (Rogers et al., 2009), and enhancing the oxidation of erythrocyte membrane lipids under hypoxia (Revin et al., 2019). Therefore, we continued to investigate the related metabolic changes in erythrocytes in HAPC.

**FIGURE 4:**
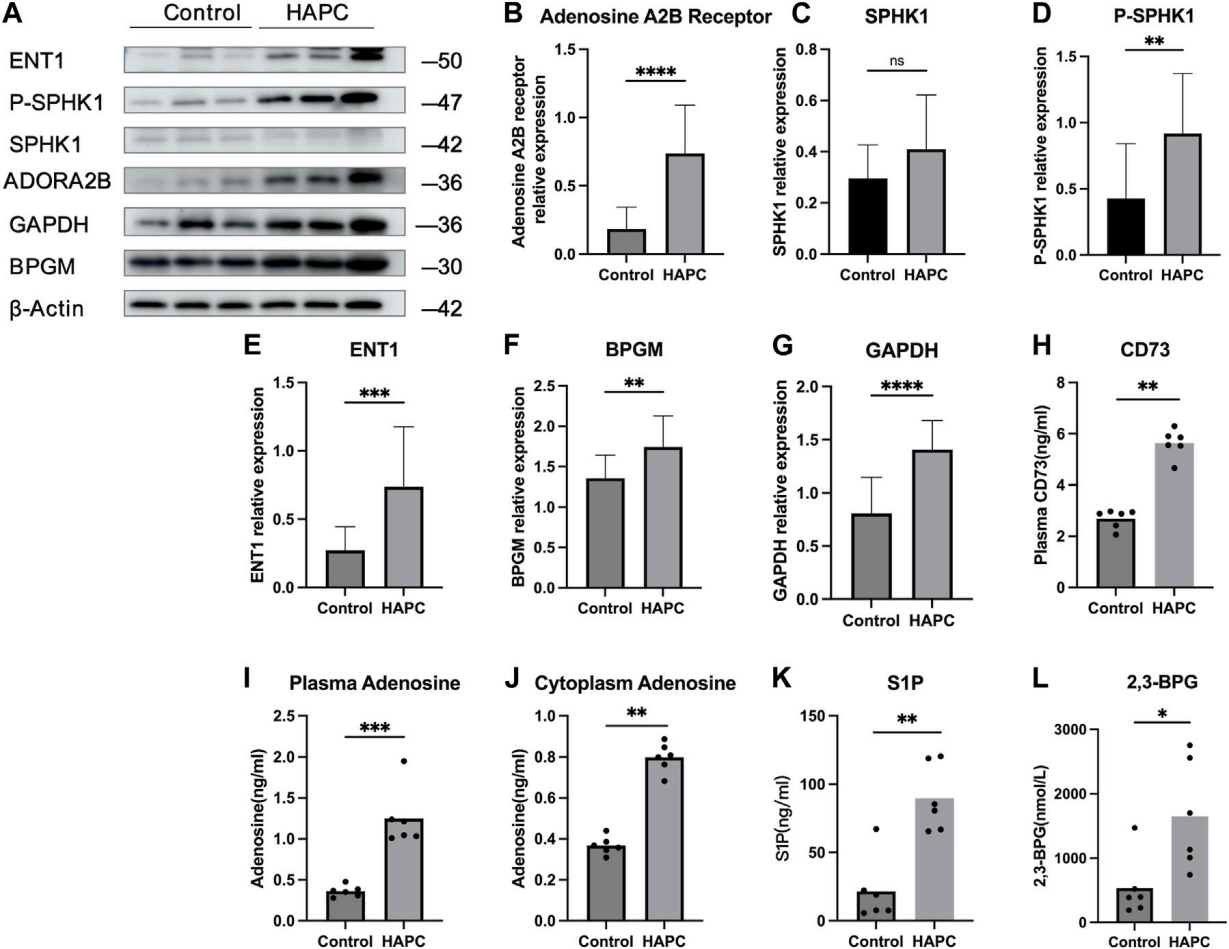
**(A)** WB display figure of erythrocyte CD73/adenosine/S1P/2,3-BPG metabolic pathway and eENT1/adenosine/BPGM/2,3-BPG metabolic pathway–related proteins. Statistical results of adenosine A2B receptor–related **(B)**, SPHK1-related **(C)**, P-SPHK1-related **(D)**, eENT1-related **(E)**, BPGM-related **(F)**, and GAPDH-related expressions **(G)**. Statistical result for plasma CD73 **(H)**, plasma adenosine **(I)**, erythrocyte cytoplasmic adenosine content **(J)**, erythrocyte cytoplasmic S1P content **(K)**, and erythrocyte cytoplasmic 2,3-BPG contents **(L)**. WB, Western blotting; CD73, 5′-nucleotidase; S1P, sphingosine-1-phosphate; BPG, 2,3-bisphosphoglycerate; eENT1, erythrocyte equilibrative nucleoside transporter 1; SPHK1, sphingosine kinase 1; P-SPHK1, phospho-SPHK1; BPGM, bisphosphoglycerate mutase; GAPDH, glyceraldehyde 3-phosphate dehydrogenase.

In people who rapidly ascend to high altitudes, erythrocyte S1P level increases steeply. Moreover, a mouse model demonstrated elevated plasma CD73 and adenosine levels. The increased adenosine binds to erythrocyte ADORA2B, which further activates SPHK1-induced S1P production, promoting glycolysis to produce a large amount of 2,3-BPG, thereby inducing oxygen release to protect tissues from hypoxia (Liu et al., 2016; Sun et al., 2016). Moreover, in sickle cell disease, the binding of adenosine to ADORA2B activates SPHK1, thereby increasing S1P production (Sun et al., 2015). Therefore, we hypothesized that the lipid metabolism-related pathways in erythrocytes are also activated to alleviate tissue hypoxia under chronic hypoxic conditions in HAPC. A previous study by our group revealed that in humans and mice under chronic hypoxia, intracellular S1P in erythrocytes is continuously elevated (Qile et al., 2023). In this study, our results showed that plasma CD73 levels in the control group were significantly lower than in the HAPC group (*p* = 0.004) (Figure 4H). Hypoxia induces an increase in CD73, promoting the conversion of AMP to adenosine, which leads to elevated levels of free adenosine. The concentration of extracellular adenosine in erythrocytes was significantly higher in the HAPC group than the control group (*p* < 0.004) (Figure 4I). Adenosine binds to ADORA2B and activates the downstream SPHK1, and the phosphorylation of SPHK1 further promotes the expression of S1P. Our results revealed that ADORA2B, phosphorylated SPHK1, and S1P levels were all increased (*p* < 0.001, *p* = 0.086, *p* = 0.004, and *p* = 0.01, respectively) (Figures 4B, C, D, K), promoting the production of 2,3-BPG by elevating the expression of GAPDH in the glycolytic pathway. The expression levels of GAPDH and 2,3-BPG were significantly higher in the HAPC group than the control group (*p* < 0.001, *p* = 0.016) (Figure 4G, L).

Recently, Chen et al. (2023) discovered that erythrocyte equilibrative nucleoside transporter 1 (eENT1) can promote the uptake of extracellular adenosine into erythrocytes, which enhances purine metabolism to activate BPGM. This process leads to increased production of 2,3-BPG, alleviating the renal oxidative stress and damage induced by severe hypoxia. In our study, we also found that under long-term hypoxia, HAPC rats exhibit higher levels of ENT1 expression and intracellular adenosine in erythrocytes than did those in the control group (*p* = 0.001, *p* < 0.001) (Figures 4E, J). Additionally, we observed a significant increase in the expression of BPGM, the key enzyme in the Rapoport–Luebering shunt, a metabolic bypass of the glycolytic pathway, in the HAPC group (*p* < 0.004) (Figure 4F). This indicates that activation of this bypass induces a further increase in 2,3-BPG. Therefore, we believe that in HAPC rats, both the CD73/adenosine/S1P/2,3-BPG and eENT1/adenosine/BPGM/2,3-BPG metabolic pathways are activated in erythrocytes (Figure 4A). The activation of these pathways represents a metabolic adaptation of erythrocytes to hypoxia, ultimately leading to the production of 2,3-BPG, which, as a specific allosteric regulator in erythrocytes, reduces the affinity of hemoglobin for oxygen and promotes the release of oxygen.

## 4 Discussion

The plateau regions of the world are vast, with a significant portion of the population residing at high altitudes (>2,500 m), primarily the Qinghai–Tibet plateau, the Ethiopian plateau, and the Andes mountains of South America. As altitude increases, both the oxygen content and atmospheric pressure gradually decrease. Some individuals living in these highlands for extended periods are unable to adapt to the low-oxygen and low-pressure environment. Chronic hypoxia can cause excessive proliferation of erythrocytes, with gradual development into HAPC. The excessive increase in erythrocytes can result in increased blood viscosity, slow blood flow, reduced oxygen-carrying capacity of erythrocytes, and disrupted microcirculation, impacting the organs and tissues of the body. Consequently, the affected population may be prone to various discomforts (such as headaches, dizziness, nausea, decreased exercise tolerance, and sleep disorders). Although HAPC is believed to be related to factors such as hypoxia-inducible factor, erythropoietin (EPO), susceptibility genes (Xu et al., 2015), an imbalance in apoptosis/proliferation, plasma ergothioneine and nitrogen oxide balance, and certain inflammatory factors (Yi et al., 2021). However, the specific molecular mechanisms remain unclear. In this study, we successfully established an HAPC rat model (Table 1), with a significant increase in peripheral blood erythrocyte count, hemoglobin content, and hematocrit. In model rats, HAPC-related erythrocyte increase was associated with enhanced bone marrow hematopoietic function and reduced erythrocyte apoptosis, whereas numerous abnormal erythrocytes, increased EOF, and reduced hemolysis resistance were associated with erythrocyte metabolism. We showed that CD73/adenosine/S1P/2,3-BPG and eENT1/adenosine/BPGM/2,3-BPG metabolic pathways in erythrocytes are activated in HAPC rats, facilitating oxygen release.

Tang et al. (2019) found that under chronic hypoxic conditions, the survival rate of peripheral blood erythrocytes increased. They also observed that chronic hypoxia could enhance the presence of CD71^+^ and Ter119^+^ monocytes in the bone marrow, affecting the proliferation, differentiation, and apoptosis rates of erythroblasts (Yu et al., 2018). However, other studies on patients with HAPC have reported a decrease in the apoptosis rate of erythroblasts in the bone marrow, contradicting these findings (Wang et al., 2018; Ma et al., 2019). This discrepancy may be attributed to different durations of hypoxic exposure in HAPC patients and mouse models, suggesting a time-dependent compensation mechanism.

Under normal conditions, the balance between Fas/FasL-mediated apoptosis and EPO-mediated proliferation maintains a stable hematopoietic environment in the body. Studies have shown that under normal conditions, mature erythrocytes are fully tolerant of Fas-mediated apoptosis, whereas FasL mediates the apoptosis of immature erythrocytes under physiological levels of EPO. However, under the hypoxic conditions of high altitude, EPO levels significantly increase while apoptosis remains steady, leading to a shift toward excessive erythrocyte proliferation. The soluble molecule sFas can also competitively bind to FasL to inhibit apoptosis (De Maria et al., 1999).

To date, no studies have investigated the apoptosis of peripheral blood erythrocytes in HAPC. Therefore, we examined the levels of RETs in bone marrow and peripheral blood as well as the apoptosis rate of peripheral blood erythrocytes. This approach offers a more direct assessment of erythrocytosis. Our findings indicated an increase in RETs in both the peripheral blood and bone marrow of HAPC rats, likely due to enhanced differentiation of erythrocyte progenitors in the bone marrow stimulated by chronic hypoxia, leading to more RETs being released into the blood to form mature erythrocytes. Additionally, our results showed a decrease in the apoptosis of peripheral blood erythrocytes, potentially prolonging their lifespan, increasing their duration of oxygen transport in the blood, and more effectively compensating for tissue hypoxia. In some diseases where there is an abnormal increase or decrease in erythrocytes, such as hereditary spherocytosis (Rey-Barroso et al., 2023), thalassemia (Joishy et al., 1986), leukemia, and malnutrition anemia (Majumder et al., 2006), morphological changes occur in erythrocytes.

Studies have suggested that factors affecting the deformability of peripheral blood erythrocytes primarily include the shape of the erythrocytes, viscosity of the cytoplasm, and mechanical properties of the cell membrane (Shin et al., 2007; Lin et al., 2014). The calcium pump on the erythrocyte membrane depends on ATP to maintain intracellular and extracellular calcium homeostasis. When intracellular ATP levels decrease or the influx of calcium exceeds the transport capacity of the calcium pump, intracellular calcium accumulation occurs. Calcium deposition on the membrane of erythrocytes leads to a loss of flexibility and adaptive capacities, making the membrane rigid and reducing its deformability. This converts the normally biconcave disc–shaped erythrocytes into vesicular erythrocytes and acanthocytes (Du and Zhao, 2008). Studies by Nie et al. (2011) found a significant increase in the concentration of calcium within erythrocytes of rats exposed to hypoxia for 28 days. Additionally, research on the erythrocyte morphological changes in populations rapidly ascending to high altitudes revealed a significant increase in the number of erythrocytes with abnormal morphologies (Gu et al., 2017). Abnormal-shaped erythrocytes are significantly correlated with oxidative stress (Gyawali et al., 2015). An increase in abnormal-shaped erythrocytes may adversely affect the stability of the erythrocyte membrane and could also impact normal erythrocyte functions. Nonetheless, few studies have been conducted on morphological changes in erythrocytes in chronic HAPC. We found a significant increase in abnormal-shaped erythrocytes in the HAPC group, predominantly vesicular erythrocytes and acanthocytes. With increased surface area compared with normal erythrocytes, vesicular erythrocytes have an enhanced ability to bind and release oxygen. Previous study has shown that in alcoholic cirrhosis and abetalipoproteinemia, acanthocytosis is accompanied by a significant increase in cholesterol levels and the proportion of sphingomyelin and glycerophospholipids (Wong, 2004). These lipids are crucial components of the erythrocyte membrane, and changes in these components may lead to membrane abnormalities. The possible mechanisms for the generation of acanthocytes include abnormal phospholipid metabolism and an increased ratio of cholesterol to phosphatidylcholine in the cell membrane, both of which can cause abnormalities in the erythrocyte membrane (Chen and Guo, 2001). These abnormalities in the membrane may in turn result in changes in deformability and oxygen-carrying capacity of erythrocytes. We consider that the generation of vesicular erythrocytes may be compensatory for erythrocytes, whereas acanthocytes may be indicative of decompensation.

In HAPC rats, an increase in EOF was observed, accompanied by decreased resistance of cells to hemolysis in hypotonic solutions. Measurements of plasma-free hemoglobin further confirmed that intravascular hemolysis was increased in these rats. However, whether the increase in EOF was solely responsible for the hemolysis remains unclear, as it may also have been related to an increase in abnormal-shaped erythrocytes and abnormalities in erythrocyte membrane metabolism. The specific mechanisms of hemolysis require further investigation. An increase in EOF in HAPC rats indicated erythrocyte membrane defects, leading to diminished resistance to hypotonic solutions and an increased tendency toward hemolysis. Additionally, membrane abnormalities are a primary contributor to intravascular hemolysis. Therefore, we hypothesized that the abundance of abnormally shaped erythrocytes and elevated hemolysis might be associated with metabolic abnormalities in erythrocytes of HAPC rats. To explore this possibility, we conducted a detailed examination of changes in erythrocyte metabolic pathways.

S1P is a biologically active signaling lipid. In erythrocytes, the absence of S1P-degrading enzymes allows these cells to produce and store substantial amounts of S1P (Igarashi et al., 2003; Ogawa et al., 2003; Le Stunff et al., 2007; Bode et al., 2010). Additionally, the activation of S1P in erythrocytes primarily depends on cytosolic SPHK1. Our results further confirmed a significant increase in levels of HAPC rat plasma CD73, which facilitates the conversion of AMP to adenosine, thereby increasing circulating adenosine. Adenosine interacts with ADORA2B, stimulating the phosphorylation of intracellular SPHK1 to increase S1P levels, promoting the generation of substantial amounts of 2,3-BPG through glycolysis.

eENT1, a crucial purinergic cellular component, regulates plasma adenosine levels in humans at high altitudes and in hypoxic mice (D’Alessandro and Xia, 2020). It also serves as the primary transporter for adenosine uptake into cells and a key regulator in “hypoxia-adaptive memory” (Song et al., 2017). Increased eENT1 expression elevates intracellular adenosine levels and BPGM expression and enhances the Rapoport–Luebering shunt in glycolysis, thereby promoting the production of 2,3-BPG. However, this study did not investigate whether the entry of adenosine into cells leads to enhanced purine metabolism and increased 2,3-BPG production.

Taken together, our findings identified the activation of both the CD73/adenosine/S1P/2,3-BPG and eENT1/adenosine/BPGM/2,3-BPG metabolic pathways (Figure 1). The potential interaction between these pathways remains unverified in this study. We believe that these metabolic pathways likely synergize to enhance 2,3-BPG production, a hypothesis we aim to investigate further in future research.

The following limitations of this study should be mentioned. First, this study was limited to an animal model of plateau erythrocytosis and did not include patients with plateau erythrocytosis for validation, which may vary among species. Second, erythrocyte mechanical fragility is a measure of erythrocyte susceptibility to mechanical stress (Ziegler et al., 2017). We neglected the ability of the erythrocytes themselves to cope with mechanical stress in both groups, which made it impossible to further explore whether erythrocyte osmotic fragility was due to a change in the erythrocytes themselves or a change in osmotic fragility during sample manipulation due to a decrease in the ability of the erythrocytes to resist mechanical stress. We can state only that the erythrocyte osmotic fragility does change, but it is unclear whether a mechanical fragility change is also involved. Third, numerous factors contribute to hemolysis, such as immunity, heredity, hypoxia, drugs, infection, and exercise (Banfi and Melegati, 2008; Lee et al., 2015; Grygorczyk and Orlov, 2017; Kawakami and Ito, 2020; Sahu et al., 2021; Shi et al., 2023). Hemolysis is categorized into intravascular and extravascular hemolysis, according to the location of erythrocyte rupture. In this study, we only briefly discussed intravascular hemolysis, and intravascular hemolysis is the result of hypoxia or mechanical damage to red blood cells (Mao et al., 2011; Mairbaurl, 2013), and we did not further explore the reasons for intravascular hemolysis, whether there is oxidative stress on red blood cells that promotes the occurrence of hemolysis.

In summary, the increase in erythrocytes in HAPC rats was associated with enhanced hematopoietic function in the bone marrow and reduced erythrocyte apoptosis. The presence of numerous abnormal-shaped erythrocytes (vesicular erythrocytes and acanthocytes), increased EOF, and decreased hemolysis resistance in HAPC rat erythrocytes are all somewhat related to erythrocyte metabolism. To adapt to the hypoxic environment, the CD73/adenosine/S1P/2,3-BPG and eENT1/adenosine/BPGM/2,3-BPG metabolic pathways are both activated in HAPC rat erythrocytes. This leads to substantial production of 2,3-BPG, the negative allosteric modulator of Hb-O_2_, which diminishes the binding affinity of hemoglobin for oxygen and facilitates oxygen release. Recognizing the activation of these two pathways enhances our understanding of the hypoxic adaptation of erythrocytes in HAPC, providing robust experimental evidence to inform future development of preventive and therapeutic strategies for HAPC. Our subsequent research will focus on exploring potential interactions between these two pathways.

## Data Availability

The original contributions presented in the study are included in the article/Supplementary Material, further inquiries can be directed to the corresponding authors.
