# Supplementary material for: Alteration in the number, morphology, function, and metabolism of erythrocytes in high-altitude polycythemia
Source: Front Physiol. 2024 Feb 15;15:1359357. doi: 10.3389/fphys.2024.1359357 (PMC10902074; doi:10.3389/fphys.2024.1359357)
Supplement: Supplementary file 1 [file Table1.DOCX]

Supplemental Table S1：Reagent and consumable information

| Name | Company | Catalog No. |
| --- | --- | --- |
| 1×TBS buffer solution（powder） | Solarbio | T1083 |
| 20×TBST buffer solution | Solarbio | T1082 |
| 10×PBS buffer solution | Solarbio | P1022 |
| 2×Protein loading buffer | Solarbio | P1019 |
| High-efficiency RIPA tissue/cell lysates | Solarbio | R0010 |
| Protease phosphatase inhibitor  mixture | Beyotime | P1045 |
| 30% acrylamide（29:1） | Solarbio | A1010 |
| 1.5M Tris-HCl buffer solution（pH=8.8） | Solarbio | T1010 |
| 1M Tris-HCl buffer solution  （pH=6.8） | Solarbio | T1020 |
| 10%SDS solution | Solarbio | S1010 |
| Ammonium persulfate | Macklin | 7727-54-0 |
| PAGE gel accelerator | Solarbio | T8090 |
| 1× Tris-Glycine electrophoresis buffer (powder) | Solarbio | T1072 |
| Weastern membrane transfer solution | Beyotime | P0021B |
| Methyl alcohol | Macklin | 67-56-1 |
| Transfer membrane | Millipore | REF:ISEQ00010 |
| Bovine serum albumin V | Solarbio | A8020 |
| Weastern Blot membrane regeneration solution (strong) | Solarbio | SW3022 |
| BD Retic-Count^TM^ Reagent | BD | REF:349204 |
| DRAQ5^TM^ | Biolegend | SKU:424101 |
| FITC anti-rat CD45 | Biolegend | 202205 |
| PE Mouse Anti-Rat CD71 | BD | 554891 |
| Annexin V-FITC/PI Apoptosis Kit | Elabscience | REF:E-CK-A211 |
| Rat Adenosine（AD）ELISA Kit | Camilo | 2R-KMLJr31257 |
| Rat 2,3-bisphosphoglycerate(2,3-BPG)  ELISA Kit | Camilo | 2R-KMLJr135384 |
| Rat Sphingosine-1-Phosphate(S1P) ELISA Kit | Camilo | 2R-KMLJr30008 |
| Rat free haemoglobin(FHb)ELISA Kit | Camilo | 2R-KMLJr31012 |
| Rat 5´Nucleotidase(5´NT/CD73)  ELISA Kit | Camilo | 2R-KMLJr31122 |
| Erythrocyte osmotic fragility Test Kit (Parpart colorimetric assay) | Solarbio | BC8010 |
| i-STAT G3+ Cartridge | Abbott | LOT:N23103A |
