# Supplementary figures and images for: Alteration in the number, morphology, function, and metabolism of erythrocytes in high-altitude polycythemia
Source: Front Physiol. 2024 Feb 15;15:1359357. doi: 10.3389/fphys.2024.1359357 (PMC10902074; doi:10.3389/fphys.2024.1359357)

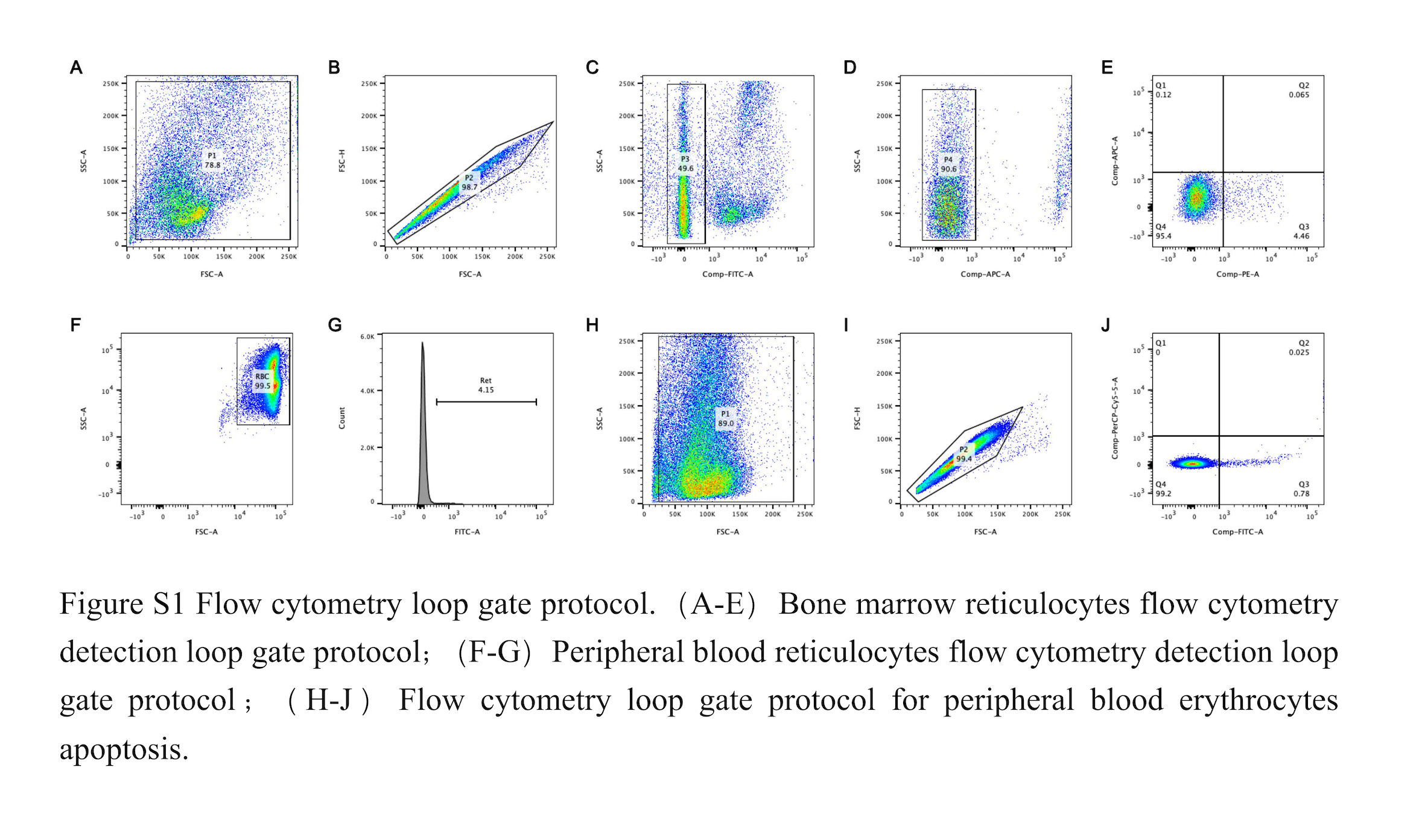

Supplement: Supplementary file 2 [file Image1.TIF]
