# Supplementary material for: Alteration in the number, morphology, function, and metabolism of erythrocytes in high-altitude polycythemia
Source: Front Physiol. 2024 Feb 15;15:1359357. doi: 10.3389/fphys.2024.1359357 (PMC10902074; doi:10.3389/fphys.2024.1359357)
Supplement: Supplementary file 3 [file Table2.DOCX]

| Supplemental Table S2 :Method of Parpart NaCI dilution | | | |
| --- | --- | --- | --- |
| Number | Parpart NaCl working fluid(ml) | ddH_2_O(ml) | NaCl concentration(g/L) |
| 1 | 4.0 | 0.0 | 10.0 |
| 2 | 3.6 | 0.4 | 9.0 |
| 3 | 3.4 | 0.6 | 8.5 |
| 4 | 3.2 | 0.8 | 8.0 |
| 5 | 3.0 | 1.0 | 7.5 |
| 6 | 2.8 | 1.2 | 7.0 |
| 7 | 2.6 | 1.4 | 6.5 |
| 8 | 2.4 | 1.6 | 6.0 |
| 9 | 2.2 | 1.8 | 5.5 |
| 10 | 2.0 | 2.0 | 5.0 |
| 11 | 1.8 | 2.2 | 4.5 |
| 12 | 1.6 | 2.4 | 4.0 |
| 13 | 1.4 | 2.6 | 3.5 |
| 14 | 1.2 | 2.8 | 3.0 |
| 15 | 1.0 | 3.0 | 2.5 |
| 16 | 0.8 | 3.2 | 2.0 |
| 17 | 0.4 | 3.6 | 1.0 |
